# Recent Clonal Origin of Cholera in Haiti

**DOI:** 10.3201/eid1704.101973

**Published:** 2011-04

**Authors:** Afsar Ali, Yuansha Chen, Judith A. Johnson, Edsel Redden, Yfto Mayette, Mohammed H. Rashid, O. Colin Stine, J. Glenn Morris

**Affiliations:** Author affiliations: University of Florida, Gainesville, Florida, USA (A. Ali, Y. Chen, J.A. Johnson, E. Redden, M.H. Rashid, J.G. Morris, Jr);; Saint Mark’s Hospital, Arbonite, Haiti (Y. Mayette);; University of Maryland College of Medicine, Baltimore, Maryland, USA (O.C. Stine)

**Keywords:** Cholera, Haiti, Vibrio cholerae, bacteria, variable-number tandem-repeat, molecular epidemiology, clonal origin, expedited, dispatch

## Abstract

Altered El Tor *Vibrio cholerae* O1, with classical cholera toxin B gene, was isolated from 16 patients with severe diarrhea at St. Mark’s Hospital, Arbonite, Haiti, <3 weeks after onset of the current cholera epidemic. Variable-number tandem-repeat typing of 187 isolates showed minimal diversity, consistent with a point source for the epidemic.

On October 21, 2010, isolation of toxigenic *Vibrio cholerae* O1 from patients with severe diarrhea was confirmed by the National Laboratory of Public Health of the Ministry of Public Health and Population in Haiti (*1*). These cases indicated onset of epidemic cholera in Haiti and were followed by rapid spread of the disease throughout the country. Illness occurred in the setting of major disruptions of water and sewage facilities resulting from the January 12, 2010, earthquake and associated deficiencies in local public health infrastructure (*1*).

Before the current epidemic, cases of cholera had not been reported in Haiti since 1960 (*2*), and disease had not spread into Haiti during expansion of the El Tor pandemic into Latin America that began in Peru in 1991. However, toxigenic *V. cholerae* O1 are present along the US Gulf Coast (*3**,**4*) and in other coastal areas in the Western Hemisphere. In conjunction with ongoing public health activities in Haiti by the University of Florida, we analyzed fecal samples from patients early in the epidemic. Data obtained on *V. cholerae* strain diversity and gene content improved our understanding of the epidemiology of this outbreak.

## The Study

Fecal samples from 19 patients were provided by staff at St. Mark’s Hospital, Artibonite, Haiti, to University of Florida investigators on November 9, 2010, <3 weeks after onset of the epidemic. Microbial testing of samples was reviewed and approved by the University of Florida Institutional Review Board. Samples were directly plated on thiosulfate citrate bile salts sucrose agar and placed in alkaline peptone water for enrichment. Yellow colonies were identified by standard biochemical tests and confirmed by PCR for the outer membrane protein W gene (*5*).

*V. cholerae* O1 Ogawa was isolated from 16 of 19 samples. All isolates were El Tor biotype and had the El Tor type regulatory gene for phage lysogeny and the co-regulated pilus A gene identified by PCR (*6**,**7*). All isolates were newly identified altered El Tor that carried the classical cholera toxin B gene, as determined by mismatch amplification mutation assay–PCR (*8*).

To evaluate genetic diversity, we randomly chose <20 colonies (average 14.4) from each of 13 fecal samples that were culture positive without enrichment. A total of 187 colonies were typed by using multilocus variable-number tandem-repeat (VNTR) analysis as described (*9**–**12*). We used 5 loci (VC0147, VC0437 [the VC0436-7 intergenic region], VC1650, VCA0171, and VCA0283) reported by Ghosh et al. (*10*) and Choi et al. (*12*). Only 9 sequence types (STs) were identified (Figure); all were within 1 clonal complex, and each differed from the others by 1 allele. Type A (8,4,6,13,36) (Figure) was the dominant ST, present in 9 of 13 patients (Table). In 6 of these 9 patients, A was the only type identified. As reported (*10*), loci on the smaller chromosome had the greatest diversity (3 alleles for VCA0171 and 5 alleles for VCA0283) compared with no variation for VC0437 or VC1650 and only 1 change in VC0147. None of these STs has been reported in studies of strains from Bangladesh, India, Vietnam, or Mozambique (*9**–**12*).

**Figure:**
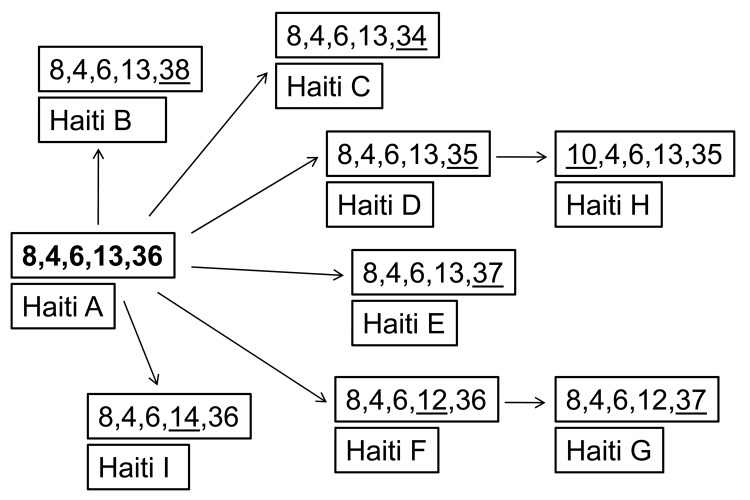
Relationship of *Vibrio cholerae* variable-number tandem-repeat sequence types from Haiti, 2010. Numbers represent number of repeats for the 5 alleles tested (VC0147, VC0436-7, VC1650, VCA0171, and VCA0283). **Boldface** indicates the ancestral sequence type; underline indicates alleles that have changed.

**Table:** Distribution of *Vibrio cholerae* variable-number tandem-repeat sequence types among 16 patients with severe diarrhea, Haiti, 2010*

Pattern	Patient no. and distribution
A = 8,4,6,13,36	P1 = 20/20, P2 = 18/18, P4 = 4/19, P6 = 1/20, P7 = 15/20, P9 = 19/19, P12 = 11/11, P17 = 1/1, P18 = 19/19
B = 8,4,6,13,38	P6 = 1/20
C = 8,4,6,13,34	P4 = 7/19
D = 8,4,6,13,35	P4 = 4/19
E = 8,4,6,13,37	P6 = 18/20, P14 = 1/1, P15 = 20/20, P20 = 1/1
F = 8,4,6,12,37	P8 = 2/18
G = 8,4,6,12,36	P8 = 16/18
H = 10,4,6,13,35	P4 = 4/19
I = 8,4,6,14,36	P7 = 5/20

*Numbers represent number of repeats for the 5 alleles tested (VC0147, VC0436–7, VC1650, VCA0171, and VCA0283). A is the dominant sequence type, identified in 9 of 16 patients for whom variable-number tandem-repeat data were available. One sequence type was found for 6 of 9 patients for whom multiple isolates were typed.

## Conclusions

Profound disruption of sanitary and public health infrastructure in Haiti resulting from the January 12, 2010, earthquake created an environment in which rapid spread of a disease such as cholera might be expected. However, because cholera had not been reported in Haiti since 1960, its sudden appearance raises questions about its origin, which has implications for understanding transmission pathways and potential for further spread.

Estuarine and freshwater/riverine (*13*) environments are well-recognized reservoirs for *V. cholerae* and show long-term persistence of epidemic strains in the absence of human cases. The US Gulf Coast is an excellent example of this environment, and periodic cholera cases occur there, likely linked to a common clonal strain that has a characteristic pulsed-field gel electrophoresis (PFGE) banding pattern (*14*). Cases along the Gulf Coast have intermittently appeared since 1978 (*3*). Indigenously acquired cholera cases linked to seafood consumption were reported in October 2005 in Louisiana in temporal association with Hurricanes Katrina and Rita (*4*).

When cholera appeared in Haiti in October 2010, cases were clustered along a 20-mile stretch of the Artibonite River, and 18 (67%) of 27 hospitalized patients reported drinking untreated water from the river or canals before illness onset, which is consistent with the river as a source of infection (*1*). Although these data are consistent with a limited origin for the epidemic, whether these strains represent a persistent clone in the river environment or a new strain in Haiti is unknown.

Epidemic strains of *V. cholerae* O1 and O139 show a high degree of genetic similarity, making it difficult to separate various subgroups by using standard molecular typing approaches such as ribotyping, PFGE, and multilocus sequence typing (*6*). PFGE types tend to change slowly and are useful primarily for distinguishing strains in different pandemics or between continental branches of pandemics (*11**,**14*). PFGE has shown that strains from Haiti are similar to strains from southern Asia and other regions (*1*). Sequences from 2 isolates from Haiti were most similar to El Tor isolates from Bangladesh in 2002 and 2008 (*15*).

In recent studies (*9**–**12*), VNTR typing has provided greater discrimination for ongoing or established epidemic O1 and O139 serogroups. Three loci (VC0147, VC0437, and VC1650) on the large chromosome were more stable (*9**–**11*) and are likely to be considered the best loci for estimating across distances, especially because of our observation of large differences in genotypes between locations 50 miles apart in rural Bangladesh (*9*).

Although genotype 8,4,6 has not been seen in other studies (*9**–**12*), genotype 9,4,6 was common in Dhaka (*11*) and associated with O1 Ogawa, the same serotype and biotype as strains from Haiti. In the absence of a more comprehensive global VNTR database, establishing a definite source for strains from Haiti on the basis of VNTR typing is not possible. However, our findings are consistent with those of others studies implicating southern Asia as the source for these strains on the basis of deletion/insertion data for the superintegron and the sulfamethoxazole/trimethoprim resistance integron island, and from analysis of single nucleotide polymorphisms, including those in the cholera toxin gene identified in the complete sequence of the strain from Haiti (*15*).

Our finding of only 9 STs differing at 1 allele among 187 colonies underscores the clonality of the strains from Haiti. Given the degree of diversity in STs among environmental strains in Bangladesh, including development of almost entirely different sets of multiple ST patterns among isolates from locations 50 miles apart, lack of diversity among isolates from Haiti would support the hypothesis that the epidemic in Haiti was caused by 1 clone that had little time to undergo diversification of STs expected of strains persistent in an environmental reservoir for extended periods.

Although altered El Tor strains have evolved only within the past several decades, which also argues against a long-standing environmental source (*8*), VNTR analysis, with its rapid molecular clock, can be used to test theories about the origin of the strain in Haiti and provide a unique opportunity to follow diversification of this clone over time and with human passage during an epidemic. This analysis also reinforces large diversity of strains seen in cholera-endemic regions, and the apparent likelihood that infections in these areas can be caused by simultaneous infections with multiple strains (*11*).
